# Development and Validation of an Algorithm for the Identification of Audible Medical Alarms

**DOI:** 10.7759/cureus.11549

**Published:** 2020-11-18

**Authors:** Paul Potnuru, Richard H Epstein, Richard McNeer, Christopher Bennett

**Affiliations:** 1 Anesthesiology, University of Texas Health Science Center at Houston, Houston, USA; 2 Anesthesiology, University of Miami Miller School of Medicine, Miami, USA; 3 Music Engineering, Frost School of Music, University of Miami, Miami, USA

**Keywords:** medical audible alarms, alarm recognition, digital signal processing

## Abstract

Audible medical alarms are ubiquitous in acute healthcare environments, but caregivers cannot reliably identify them. Furthermore, background noise and psychoacoustic factors can interfere with alarm recognition and contribute to alarm fatigue. We developed and validated an acoustic digital signal processing algorithm for the automatic identification of audible medical alarms. The algorithm uses the short-time Fourier transform to decompose audio signals and extract the alarm sounds' fundamental frequencies, harmonics, and periodicity. This information is then used to classify and recognize these sounds. The identification algorithm demonstrates robust performance (F1 score of 93% to 100%) and 100% negative predictive value in identifying single or multiple medical audible alarms under both quiet and noisy conditions. The algorithm we developed represents a robust approach for the identification of audible medical alarms that perform with high accuracy in noisy environments. It can be used to identify and classify alarms in medical settings for research and clinical purposes.

## Introduction

Audible medical alarms are ubiquitous in acute healthcare environments, such as the operating room and the intensive care unit. However, caregivers cannot reliably identify currently used alarm sounds [1-5]. Exposure to multiple alarms over time and the presence of background noise can further complicate the identification process. Such acoustical interference can adversely affect patient safety by increasing the risk of alarm fatigue in caregivers [6]. In this technical report, we present our work in developing digital signal processing software for the automatic recognition of audible medical alarms under conditions with both low and high levels of ambient noise. This software can serve as a research tool in studies investigating alarm fatigue in healthcare environments and as a clinical tool for integrating alarm sounds from multiple sources.

Previous studies of automatic audible alarm recognition have been primarily in the settings of industrial and traffic alarms [7-11]. Approaches described in the literature have included sinusoidal modeling, machine learning, longest common sequence identification, and amplitude-based periodicity detection [12-14]. However, these methods' accuracy has generally been limited, and their performance under noisy conditions is poor. In our approach, we took advantage of the predictable structure of the majority of current medical audible alarm sounds, as specified in IEC 60601-1-8 [15], which includes a fundamental frequency and harmonics, combined with a characteristic periodicity (repeat interval) to create an algorithm for the recognition of audible medical alarms.

## Technical report

Alarm sound selection and acquisition

We collected and analyzed 14 different alarm sounds from nine medical devices used in the OR, post-anesthesia care unit, and intensive care unit (Table 1, https://drive.google.com/drive/folders/1P1gFwsP8y12pLaYdsWeoN2FEOfMbK3gH?usp=sharing). The selected devices were a convenience sample of devices available at our institution that produced alarms spanning a wide range of frequencies and patterns. The devices used for testing included physiologic monitors, ventilators, medication and supply dispensing systems, intermittent pneumatic compression pumps, electrosurgery units, infusion pumps, and other common hospital equipment. Audio samples were recorded in the waveform audio file format (WAV) at a sampling frequency of 44.1 kHz using Audio Recorder v2.01.33 (Sony Mobile Communications, Lund, Sweden). The alarm sounds were analyzed individually, and then a database containing the representative frequencies and periodicity of each alarm sound was created. We developed an algorithm that analyzed an input audio signal to identify alarm sounds matching our database and tested it with pure alarm sounds recorded in a quiet environment and with varying levels of background noises (https://drive.google.com/drive/folders/1OfUmo93p8uyEuWWIc5pWJztaJ6OLiZ5s?usp=sharing). Synthetic sounds used for testing were created by digitally summing the alarm sound and background noise audio files. All analyses were performed using MATLAB version R2017a and the DSP System Toolbox™ (MathWorks, Inc., Natick, MA).

**Table 1:** Device Alarms Used in Our Testing

Alarm Sound	Frequencies (Hz)	Periodicity (sec)	Autocorrelation Peak Width (sec)
Aisys CS2 Ventilator - Critical	398, 1195, 2003	0.49	0.06
Aisys CS2 Ventilator - Warning	398, 1195, 2003	0.366	0.178
Alaris PC 8015 IV Pump	2196	2.06	
BD Pyxis Medication Station	883	1.1	
Braun Outlook 400 IV Pump - Alarm	528, 1572, 2616	3.68	
Braun Outlook 400 IV Pump - Starting	786, 2347	1.05	0.1
Flowtron SCD Pump	2713	0.46	0.1
GE Carescape B650 Monitor - Warning	441, 1187	2.75	
GE Carescape B650 Monitor - Critical	506, 1497	1.02	
Megadyne Electrosurgical Unit	2315	0.64	
Omnicell (Supply Cabinet) - Door Open	2024, 2261	1	
Omnicell Medication Dispensing System	700, 1400, 1766	0.325	0.15
Philips Intellivue MP30 Monitor - Warning	485	2.104	0.24
Philips Intellivue MP30 Monitor - Medium	485, 2401	2.091	0.5

Feature extraction

**Figure 1:**
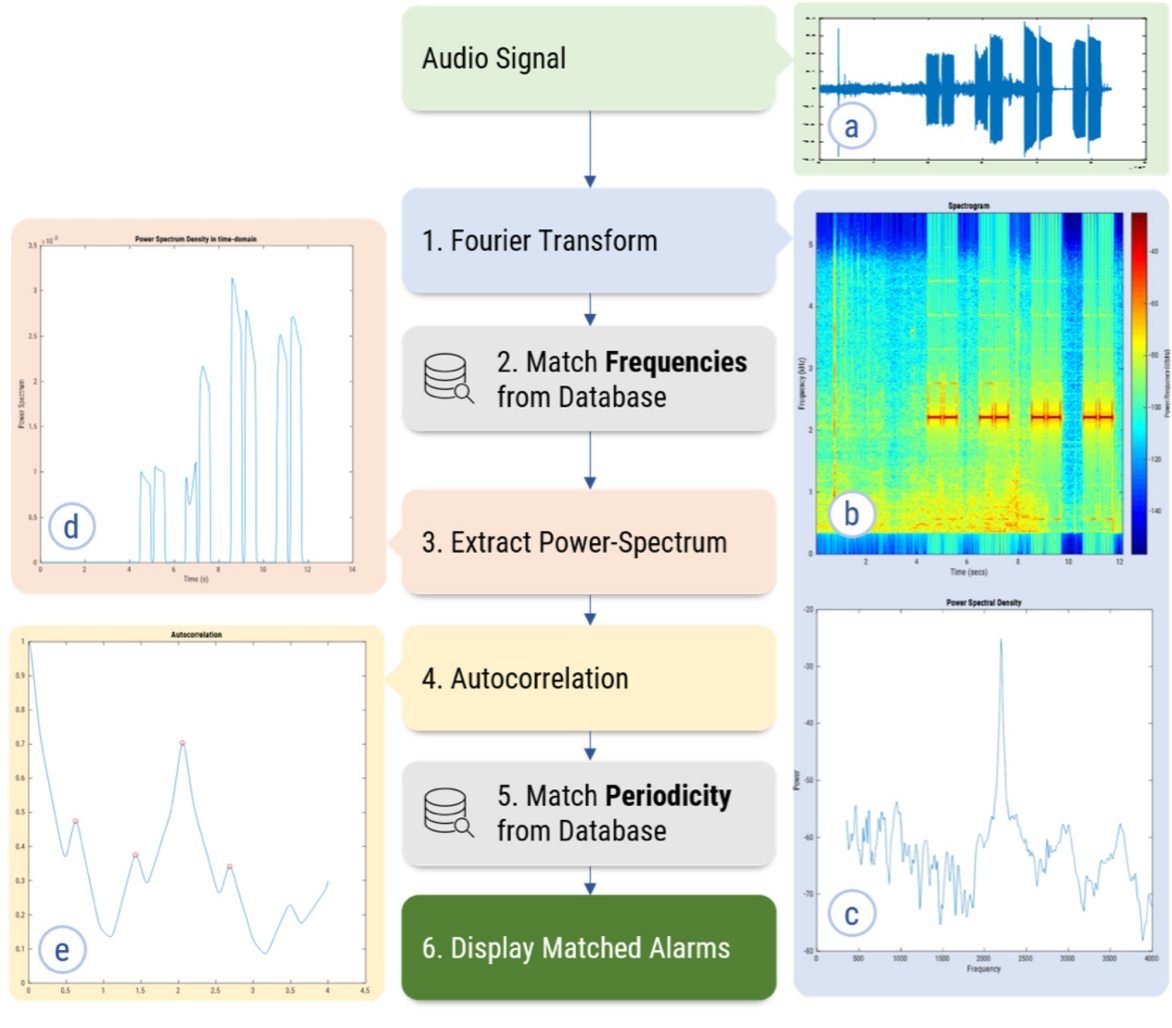
Process Diagram of the Recognition Algorithm The example is for an Alaris PC 8015 intravenous pump alarm (CareFusion, San Diego, CA). Panel (a) is a plot of the raw audio signal with time on the horizontal axis and amplitude on the vertical axis. Panel (b) shows the results of the STFT plotted on a spectrogram with increasing time on the horizontal axis, increasing frequency on the vertical axis, and the signal power (magnitude) color-coded by the scale shown on the right. Panel (c) is the PSD as a function of frequency showing a prominent peak at 2196 Hz. Panel (d) is a log-compressed power spectrum as a function of time for the frequency of interest (2196 Hz). Panel (e) is a plot of the autocorrelation of the power spectrum density in the time domain with peaks (red circles), showing a short period (first peak) at approximately 0.6 seconds. The extracted features from the sound waveforms are then matched with the database, and the source of the alarm is determined.

We began our analysis with pure alarm sounds (Figure 1a), recorded with minimal background noise. Based on the fundamental frequencies of the alarms we collected, we limited the analysis from 350 Hz to 4000 Hz. The digital audio signal was downsampled by a factor of four to eliminate higher frequency components and processed by a digital high-pass finite impulse response (FIR) filter (300 Hz stopband, 350 Hz passband) to eliminate lower frequency components. A short-time Fourier transform (STFT) of the filtered signal was computed by calculating a 256-point discrete Fourier transform (DFT) using a Hamming window of 1024 samples with an overlap of 1008 samples. This process produces a graphical representation that allows simultaneous visualization of spectral components in both time and frequency domains (Figure 1b). The results were also plotted to show the power spectral density (PSD) of the entire audio sample as a function of frequency (Figure 1c). Up to five frequencies containing the highest power for each alarm signal were identified. The additive log-compressed power spectrum of the identified frequencies was plotted against time (Figure 1d). Autocorrelation (i.e., the signal's correlation with a time-shifted copy of itself) was performed on the PSD to identify the signal's periodicity. The first peak of the autocorrelation corresponds to the alarm sound's shortest repeat interval (Figure 1e). This short interval was stored in the database along with the identified frequencies. For some alarm sounds, the width of the autocorrelation peak corresponded to the length of each tone in a repeated alarm signal. For these alarms, the autocorrelation peak width was also stored in the database. The final feature set for each alarm sound comprised a set of up to five frequencies, a short periodicity, and an autocorrelation peak width (when applicable). Thus, each alarm was characterized by a template comprising between two and seven values.

Identification algorithm

Within sample sound files, potential alarms were identified by extracting the acoustic features from those files and attempting to match them, within a tolerance range, with one or more of the alarm templates in the database. The process for recognizing alarm sounds from a sound file containing an unknown alarm sound started similarly to the pure alarm feature extraction process: Frequency components outside the range of interest were eliminated, and the STFT of the signal was computed. Then, the algorithm iterated through each alarm sound stored in the database to determine whether its frequencies, periodicity, and peak width were present in the unknown audio signal.

The algorithm first determined whether the specified frequencies for an alarm sound were prominent in the PSD using a minimum threshold of 50% of the maximum PSD. After matching the prominent frequencies, the algorithm generated a list of possible devices that produce alarms contained in the unknown audio signal. For each of these potential devices, an autocorrelation was performed on the power spectrum magnitudes of the frequencies of interest to determine the periodicity. The first peak of the autocorrelation was identified using a peak threshold of 50% of the maximum autocorrelation signal and a minimum peak distance of 150 msec. If the calculated periodicity was within 37.5 msec of the defined periodicity in the database, it was considered a positive match. The thresholds for positive identification of prominent frequencies and periodicity were determined by testing the training dataset with combinations of PSD thresholds varying from 40% to 60% in 5% increments and periodicity thresholds from 30 to 50 msec in 2.5 msec increments. Accuracy of recognition was computed using the F1 score. The combination with the highest F1 score was selected as the final threshold: 50% of the maximum PSD and 37.5 msec.

Testing protocol

For each unique alarm sound in our database, we collected five separate sound samples for the testing dataset. These sounds were separate from those used for the training dataset. Each sample was recorded at separate times and contained at least two bursts of the alarm sound. First, the sounds from the testing set were analyzed by the algorithm without any background noises (pure alarm sounds). Then, we added three different background noises to the alarm sounds to obtain a root-mean-square (RMS) signal-to-noise ratio (SNR) ranging from +6 dB to -6 dB with a step size of 1 dB in each iteration. The background noises included: (i) randomly generated pink noise, (ii) a random clip of a soundscape containing real-world operating room sounds [5], and (iii) a random clip from a royalty-free jazz music sample (available online). From the results of the alarm recognition algorithm in these scenarios, we calculated the true positive (TP), false positive (FP), and false negatives (FN) rates and computed the recall (sensitivity, TPTP + FNTPTP + FN), precision (positive predictive value, TPTP + FPTPTP + FP), and F1 score of our algorithm for each scenario. A FP in these testing scenarios was defined as identifying an alarm matching one of the templates when the alarm was not present in the sample. The F1 score, commonly used in algorithm analysis as an overall assessment of the accuracy of a categorizer, is calculated as the harmonic mean of the precision and the recall (2 × (precision × recall) precision + recall2 × (precision × recall) precision + recall). Additionally, we ran the testing protocol with a 10-second long silent WAV file (no alarm sound) to determine the negative predictive value of our algorithm (NPV, TNTN + FNTNTN + FN).

Threshold performance testing

To validate the performance of our algorithm in detecting the alarms within the specified frequency and periodicity thresholds, we performed threshold testing on two alarms with a high fundamental frequency (Flowtron SCD Pump; 2713 Hz) and a low fundamental frequency (Philips Intellivue MP30 Monitor - Warning Alarm; 485 Hz). This testing was done to assess potential issues with ambiguous identification resulting from alarm sounds from devices that might only vary slightly different from an alarm mapped in the database. For each of these alarms, we generated alarm tones as a WAV file with frequencies ranging from ±7 Hz of the alarm’s fundamental frequency with a step size of 1 Hz and periodicities ranging from ±50 msec of the alarm’s periodicity with a step size of 10 msec. This process resulted in a set of 176 generated tones for each of the two selected alarm sounds. We then ran our detection algorithm on these 352 generated alarm tones.

Results

We tested five separate recordings of 14 alarm sounds from nine different medical devices for a total of 70 alarm sound clips (N = 70). The recall ranged from 94% to 100% (Table 2) over an SNR of -6 dB (i.e., the noise is 2x louder than the signal) to +6 dB (i.e., the signal is 2x louder than the noise). The precision ranged from 93% to 100% (Table 2). The lowest F1 score was 0.957. These results indicate that the algorithm should function well in an operating room environment where ambient noise levels are frequently at least as loud as the alarm. There were no false positives over the entire range of testing conditions with various levels of noise and background sounds added in the absence of any alarms, yielding a negative predictive value of 100%. These findings indicate that the algorithm is extremely unlikely to detect that an alarm is present when, in fact, it is absent.

**Table 2:** Performance of the Algorithm in Various Background Noise Conditions SNR, signal-to-noise ratio; TP, true positive; FP, false positive; FN, false negative.

BG Noise Type	SNR (dB)	TP	FP	FN	Recall	Precision	F_1_ Score^a^
Pure Tone	No noise added	70	0	0	100%	100%	1.000
Pink Noise	-6	67	0	3	96%	100%	0.978
Pink Noise	-5	67	1	3	96%	99%	0.971
Pink Noise	-4	69	0	1	99%	100%	0.993
Pink Noise	-3	66	0	4	94%	100%	0.971
Pink Noise	-2	68	0	2	97%	100%	0.986
Pink Noise	-1	69	0	1	99%	100%	0.993
Pink Noise	0	70	0	0	100%	100%	1.000
Pink Noise	1	70	0	0	100%	100%	1.000
Pink Noise	2	70	0	0	100%	100%	1.000
Pink Noise	3	70	0	0	100%	100%	1.000
Pink Noise	4	70	0	0	100%	100%	1.000
Pink Noise	5	69	0	1	99%	100%	0.993
Pink Noise	6	70	0	0	100%	100%	1.000
OR Soundscape	-6	67	3	3	96%	96%	0.957
OR Soundscape	-5	66	2	4	94%	97%	0.957
OR Soundscape	-4	67	3	3	96%	96%	0.957
OR Soundscape	-3	68	1	2	97%	99%	0.978
OR Soundscape	-2	68	4	2	97%	94%	0.958
OR Soundscape	-1	67	2	3	96%	97%	0.964
OR Soundscape	0	70	2	0	100%	97%	0.986
OR Soundscape	1	70	4	0	100%	95%	0.972
OR Soundscape	2	69	1	1	99%	99%	0.986
OR Soundscape	3	69	0	1	99%	100%	0.993
OR Soundscape	4	70	2	0	100%	97%	0.986
OR Soundscape	5	69	0	1	99%	100%	0.993
OR Soundscape	6	70	2	0	100%	97%	0.986
Jazz Music	-6	69	5	1	99%	93%	0.958
Jazz Music	-5	69	3	1	99%	96%	0.972
Jazz Music	-4	70	4	0	100%	95%	0.972
Jazz Music	-3	70	0	0	100%	100%	1.000
Jazz Music	-2	68	1	2	97%	99%	0.978
Jazz Music	-1	69	1	1	99%	99%	0.986
Jazz Music	0	70	0	0	100%	100%	1.000
Jazz Music	1	69	3	1	99%	96%	0.972
Jazz Music	2	70	2	0	100%	97%	0.986
Jazz Music	3	70	0	0	100%	100%	1.000
Jazz Music	4	70	0	0	100%	100%	1.000
Jazz Music	5	70	1	0	100%	99%	0.993
Jazz Music	6	70	1	0	100%	99%	0.993
Cumulative	-6	203	8	7	97%	96%	0.964
Cumulative	-5	202	6	8	96%	97%	0.967
Cumulative	-4	206	7	4	98%	97%	0.974
Cumulative	-3	204	1	6	97%	100%	0.983
Cumulative	-2	204	5	6	97%	98%	0.974
Cumulative	-1	205	3	5	98%	99%	0.981
Cumulative	0	210	2	0	100%	99%	0.995
Cumulative	1	209	7	1	100%	97%	0.981
Cumulative	2	209	3	1	100%	99%	0.991
Cumulative	3	209	0	1	100%	100%	0.998
Cumulative	4	210	2	0	100%	99%	0.995
Cumulative	5	208	1	2	99%	100%	0.993
Cumulative	6	210	3	0	100%	99%	0.993

**Figure 2:**
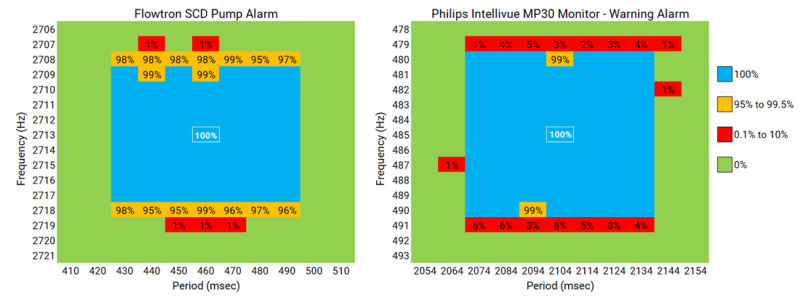
Results of the Threshold Performance Testing From Generated Alarm Tones The panel in the left shows the results for the Flowtron SCD Pump alarm, which has a fundamental frequency of 2713 Hz and a periodicity of 460 msec. The panel in the right shows the results for the Philips Intellivue MP30 Monitor - Warning Alarm, which has a fundamental frequency of 485 Hz and a periodicity of 2104 msec. Frequency is plotted on the vertical axis, centered around the fundamental frequency of the alarm sound. Periodicity is plotted on the horizontal axis, centered around the true periodicity of the alarm sound. The blue box with the white border represents the true fundamental frequency and periodicity of the alarm sound. The blue-shaded area represents 100% recall, the yellow-shaded area represents 95%–99% recall, the red-shaded area represents 0.1%–10% recall, and the green-shaded area represents 0% recall.

The threshold performance testing (Figure 2) showed 100% recall in the ±3 Hz frequency range and the ±30 msec periodicity range. There were no false positives outside the ±6 Hz frequency range and the ±40 msec periodicity range. These findings indicate that the algorithm has an excellent ability to discriminate between alarms produced by the target device and potentially a very similar alarm from a different device.

## Discussion

The algorithm we developed represents a novel approach for the identification of audible medical alarms that performs with high accuracy in noisy environments. The front end of our development process, the capture of the audio signal, worked well on a smartphone platform, followed by the transfer of the sound files for remote processing and identification. Direct transfer of the audio recording would be problematic in the medical environment due to privacy concerns. However, the MATLAB code we developed can be deployed directly to smartphones running iOS or Android, and current generation devices have sufficient computing power to both perform the digital processing and run the recognition software locally. Alternatively, the algorithms could be incorporated within other medical devices, such as physiologic patient monitors, to identify alarms produced by external devices. The amount of information in the database necessary to identify each alarm is small, consisting of only the fundamental frequencies and the periodicity of the signal; this could easily be maintained and updated on the local device as new alarms are added. Alternatively, new alarms could be adaptively added to the local device. In contrast to our algorithm, although song recognition software (e.g., Shazam, Apple, Cupertino, CA) also does local signal processing of the audio signal, the extracted features are transmitted centrally for matching, then return the artist and the name of the song [16]. In our software, the entire recognition process can be done locally, obviating the need for an internet connection during use.

The audible medical alarm algorithm we present here is reliable and expandable. One potential research use would be to facilitate studies of provider behavior in response to device alerts. For example, the algorithm could be adapted to record the length of time that various alarms remained active until addressed (i.e., response time). Such an approach could be used to study alert fatigue. A potential clinical use would be to incorporate the algorithm into a stand-alone device or within another medical device (e.g., a patient monitor or anesthesia machine) to analyze alarms in healthcare environments and notify the provider as to the potential source. This would be especially useful when the sound produced is difficult to localize or is from an unfamiliar device.

There are some limitations to our approach. First, we studied a relatively small number of alarms limited to the devices used at our institution. However, these alarm sounds spanned a wide range of frequencies and periodicities, supporting the generalizability of our algorithm. Furthermore, the results of our discrimination testing indicate that the algorithm can distinguish between alarms with very similar frequencies (7 Hz different) and repeat patterns (31 msec different). Second, the utility of the algorithm relies on the compilation of a representative database of sound templates for alarm identification. Given the ease of recording and transferring new alarm sounds using smartphones, a crowdsourcing approach to expand such a database is feasible. Third, our algorithm relies on a well-defined alarm structure with repeating tones of fixed frequencies. Although this structure represents most current audible medical alarms, the algorithm may not be applicable to alarms with more complex acoustic structures and features. Finally, under circumstances where, by happenstance, two manufacturers picked nearly the same alarm sound and pattern for different devices that were represented in the target database, there would be two potential matches presented by the algorithm. For example, if an alert from a patient warming device was within the algorithm's discrimination tolerance for an alert from an electrosurgical unit (ESU), the algorithm would report back that there was an alert from either the warmer or the ESU. However, if the warming device alert sound was present in the database but the ESU alert sound was missing, the source would be misidentified as coming from the warmer when the alarm actually came from the ESU.

## Conclusions

We developed a robust approach to identify audible medical alarms using the frequency and repeat interval that is accurate and reliable even under noisy conditions. The capabilities of the identification algorithm can be easily expanded by adding additional alarm sounds to the database. The simplicity of the approach makes it highly amenable to future adoption.
